# Health status of poor, older urban adults compared with key health indicators from the 2023 Korea National Health and Nutrition Examination Survey in the Republic of Korea: a cross-sectional comparative study

**DOI:** 10.7586/jkbns.25.086

**Published:** 2026-02-20

**Authors:** Joo Hyun Kim, Yeon Jeong Heo, Curie Ahn, Ho Young Lee, Bumjo Oh, Jae Bok Kwak, Samil Park, Jung Sik Lee, Soyeon Kim, Chaewon Nam, Taerim Lee

**Affiliations:** 1 Department of Nursing, Kangwon National University, Chuncheon, Korea; 2 Department of Nursing, Research Institute of Nursing Science, Hallym Polytechnic University, Chuncheon, Korea; 3 Raphael Nanum Foundation, Raphael International, Seoul, Korea; 4 Department of Nuclear Medicine, College of Medicine, Seoul National University, Seoul, Korea; 5 Department of Family Medicine, Seoul National University College of Medicine, Seoul, Korea; 6 Department of Statistics & Data Science, Korea National Open University, Seoul, Korea

**Keywords:** Aged, Health status disparities, Poverty, Socioeconomic factors, Urban population

## Abstract

**Purpose:**

This study compared key health indicators of poor, older urban adults attending a free clinic with those of the general older population, using data from the 2023 Korea National Health and Nutrition Examination Survey (KNHANES).

**Methods:**

This cross-sectional comparative study included 60 adults aged ≥60 years who attended the Raphael Nanum Homeless Clinic in Seoul. Participants completed a questionnaire, underwent anthropometric assessment, and provided fasting blood samples for measurement of total cholesterol, low density lipoprotein (LDL)-cholesterol, and triglycerides (TG). Obesity, current smoking, monthly alcohol use, poor self-rated health, and strength exercise (≥ 2 days/week) were defined according to 2023 KNHANES criteria and compared with age-matched 2023 KNHANES estimates for adults aged ≥ 60 years using independent t-tests and two-proportion z-tests.

**Results:**

Participants were predominantly men (80.0%) with a mean age of 79.9 years; 70.0% reported no regular income, and 46.7% rated their health as poor. Compared with their 2023 KNHANES counterparts, the clinic group had a higher prevalence of obesity (50.0% vs. 35.0%), particularly among men, and a more atherogenic lipid profile characterized by higher LDL-cholesterol despite similar total cholesterol levels and lower TG. The prevalence of current smoking (3.3% vs. 10.6%) and monthly alcohol use (31.7% vs. 53.0%) was significantly lower, whereas participation in strength exercise was low in both groups, with no significant differences observed.

**Conclusion:**

Poor, older urban adults exhibited multidimensional health disparities, including obesity, adverse lipid profiles, and markedly poorer self-rated health, despite lower levels of smoking and alcohol consumption. Community-based interventions targeting nutrition, physical activity, and chronic disease management are needed to reduce health inequalities in this vulnerable population.

## INTRODUCTION

Despite Korea’s economic growth, poverty among older adults remains severe, resulting in substantial health disparities [1]. In particular, poor, older urban adults, referred to as a medically vulnerable population, are at increased health risk due to insufficient income and limited social support [1]. Korea has the highest old-age poverty rate among Organization for Economic Co-operation and Development (OECD) countries, reaching 43.8% in 2016 and remaining around 40% in 2020, nearly three times the OECD average [2]. These low-income older adults are vulnerable to chronic diseases and have shorter life expectancy because of unbalanced diets and restricted access to healthcare [1,3].

Nutritional imbalance is a critical health factor among poor, elderly urban people. Approximately 5% of older Koreans reported the inability to consume sufficient food [4]. Food insecurity is particularly prevalent among single-person elderly households, which often results in protein and micronutrient deficiencies and leads to an increased risk of chronic diseases [5]. An earlier analysis of the Korea National Health and Nutrition Examination Survey (KNHANES) data showed that nutrient intake among low-income older adults was significantly lower than recommended levels, with poor nutritional status scores [5]. Such nutritional imbalances contribute to underweight, weakened immunity, obesity, and dyslipidemia as additional burdens [4,6].

In terms of health behaviors, older low-income adults are more disadvantaged in smoking, alcohol use, and lack of physical activity [6,7]. The exercise rates are markedly lower in older low-income adults compared to higher-income groups, and the socioeconomic gap has been widening [8,9]. While smoking rates are relatively low among older women, smoking prevalence remains high in low-income older men, placing them at considerable risk of respiratory diseases [10]. Similarly, alcohol consumption is higher among socially vulnerable older men [11].

The prevalence of chronic diseases, such as hypertension, diabetes, and hyperlipidemia, is also higher among poor urban elderly individuals compared to the general population, and comorbidities are common [6,11,12]. A higher prevalence of eight chronic diseases, including stroke, heart failure, chronic respiratory diseases, and depression, was reported among medical aid beneficiaries compared to health insurance enrollees, with more frequent hospitalizations and medical service utilization [11]. Income-related disparities in hypertension and diabetes persist [12]. These older adults also experience significant unmet healthcare needs due to financial burdens, transportation barriers, and information gaps, which were further exacerbated during the coronavirus disease 2019 (COVID-19) pandemic [13,14].

Subjective health status is also reported to be lower among low-income older adults. These individuals are more likely to rate their health as poor compared to older adults in the general population [3,15], with greater vulnerability observed among those who are older, female, or living alone [16]. These differences highlight gender-specific health vulnerabilities, with men more affected by chronic diseases and health behaviors, and women more affected by social isolation and mental health [3].

The Raphael Nanum Foundation, established in June 2015, is a humanitarian organization grounded in the value of respect for human dignity and committed to providing medical support for medically underserved populations and global health initiatives in resource-limited countries. The Foundation inherits the values of the Raphael Clinic, a free medical service for migrant workers launched in 1997, and Raphael International, established in 2007 for international health cooperation in Southeast Asia. The Foundation seeks to generate synergy through the integration of these two institutions. When public hospitals were designated as COVID-19 centers during the pandemic, the Foundation continuously provided free medical care to marginalized homeless populations who were excluded from healthcare access. As part of these activities, the Raphael Nanum Homeless Clinic provides basic medical services and health counseling for urban poor and homeless individuals, serving as an important field resource for understanding the health status of vulnerable older adults.

Thus, data from the Raphael Nanum Homeless Clinic provide valuable insight into the health conditions of poor, older urban adults. In light of the unmet needs reported by free-clinic visitors [17], this study aimed to compare serum lipid profiles and key health indicators among poor, older urban adults aged ≥ 60 years with age-matched estimates from the 2023 KNHANES. The indicators included obesity, current smoking, monthly alcohol use, poor self-rated health, and strength exercise (≥ 2 days/week). We additionally assessed whether these differences varied by sex. The findings are intended to inform targeted health promotion and support strategies for medically vulnerable older adults.

## METHODS

### 1. Study design

This study was designed as a cross-sectional comparative study to examine health disparities by comparing serum lipid profiles and key health indicators between poor, older urban adults attending a free clinic and age-matched estimates from the 2023 KNHANES.

### 2. Participants

**Figure 1.**
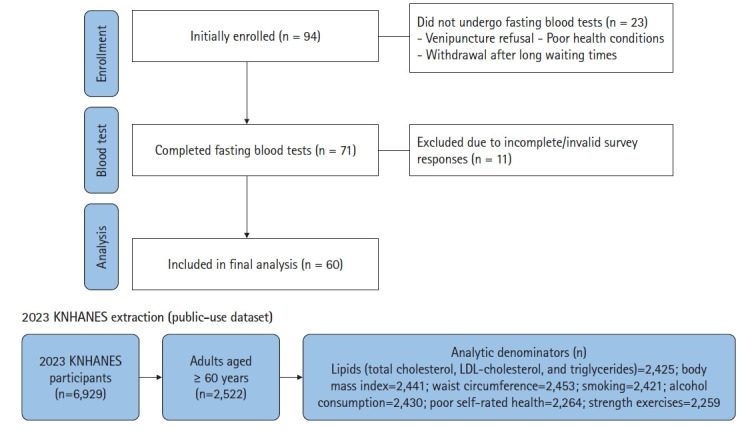
Participant flow diagram for the ﻿Raphael Nanum Homeless Clinic. KNHANES = Korea National Health and Nutrition Examination Survey.

The study participants were poor urban adults aged 60 years and older who attended the Raphael Nanum Homeless Clinic. For external comparisons, adults aged ≥ 60 years were extracted from the 2023 KNHANES, a nationally representative health survey conducted by the Korea Disease Control and Prevention Agency, using the public-use dataset. Figure 1 summarizes the participant selection flow for the Raphael Nanum Homeless Clinic sample and the extraction flow for the 2023 KNHANES comparator (aged ≥ 60 years), including indicator-specific analytic denominators. Because 2023 KNHANES analytic denominators vary by indicator across survey components (interview, examination, and laboratory measures), the 2023 KNHANES denominator used for each outcome is reported in the corresponding comparison tables. A total of 94 individuals were initially enrolled. Of these, 23 did not undergo fasting blood tests due to venipuncture refusal, poor health conditions, or withdrawal after long waiting times. Among the remaining 71 participants, 11 were excluded because of incomplete or invalid survey responses. Therefore, data from 60 participants were included in the final analysis.

The target sample size was set at 128 participants. An a priori power analysis was conducted using G*Power 3.1 [18] based on Cohen’s approach for a two-tailed independent-samples t-test (difference between two independent means). Assuming a medium effect size (d = 0.4), a significance level of 0.05, and a power of 0.95 (allocation ratio = 1:1), the minimum required sample size was 102. The target sample size was increased to 128 to account for an anticipated 20% attrition rate. Although the final analytic sample did not reach the target size, the exclusions primarily reflected unavoidable circumstances related to blood collection and survey completeness.

### 3. Instruments

Measurement tools were developed by referring to 2023 KNHANES items pertaining to the health survey, examinations, and nutrition. The variables were categorized into (1) general characteristics, (2) physiological health indicators, and (3) health indicators. All variables were coded according to predefined operational definitions, and missing responses or incomplete tests were treated as missing values. To benchmark the general older population, 2023 KNHANES estimates for adults aged ≥ 60 years were used as an external comparator. However, because publicly available 2023 KNHANES summaries do not allow reconstruction of a clinic-defined impoverished subgroup using the same set of sociodemographic variables, 2023 KNHANES-based general characteristics for an “impoverished older adult” subgroup were not presented in Table 1. In addition, 2023 KNHANES analytic denominators vary by indicator across survey components; therefore, the 2023 KNHANES denominator used for each outcome was reported in the corresponding comparison tables.

#### 1) General characteristics

General characteristics were assessed using a structured questionnaire, including 16 variables. Sex (men/women), age (continuous), education (none, elementary, middle school, high school, and ≥ college), religion (yes/no), income status (yes/no), monthly income (continuous), housing status (yes/no), cohabitation with family (yes/no), receiving basic livelihood security (yes/no), and the type of health insurance (national health insurance/medical aid) were included.

Subjective health status was measured on a 5-point Likert scale, with responses ranging from “very good (1)” to “very poor (5).” For descriptive purposes, the responses were categorized as “good” (very good/good), “fair,” and “poor” (poor/very poor). For analysis, responses were dichotomized into 1 (poor/very poor) and 0 (all others). Smoking status was categorized as current smoker (daily/occasional), former smoker, and nonsmoker, with current smoking coded as 1. Alcohol use was defined as drinking at least once per month in the past year. Strength exercise was defined as practicing muscle-strengthening exercises for ≥ 2 days per week during the past week. Body mass index (BMI, kg/m²) was calculated from weight (kg)/height (m²), and waist circumference (cm) was measured horizontally between the lower rib margin and iliac crest.

#### 2) Physiological health indicators

Blood samples were collected and analyzed according to standard laboratory protocols. Lipids (total cholesterol [mg/dL], low density lipoprotein-cholesterol [LDL-C, mg/dL], and triglycerides [TG, mg/dL]) were included. These lipid measures were selected as core indicators of dyslipidemia and cardiovascular risk and because they allow direct comparison with the 2023 KNHANES data. Given the final sample size (n = 60), including multiple additional biochemical markers risked reducing statistical power. Therefore, only the three indicators with high clinical relevance, comparability, and data completeness were selected. Other biomarkers (e.g., high density lipoprotein-cholesterol levels and liver and kidney function tests) were collected but not included in the analysis.

#### 3) Health indicators

Health indicators were calculated in accordance with the KNHANES definitions: (1) obesity prevalence (BMI ≥ 25 kg/m², Asian criteria), (2) current smoking rate, (3) alcohol use (≥ 1 time/month in the past year), (4) poor self-rated health (reported as “poor” or “very poor”), and (5) strength exercise practice frequency (≥ 2 days/week in the past week). Applying the same definitions as the KNHANES ensured comparability between poor, elderly urban adults and the general elderly population.

Primary outcomes included total cholesterol, LDL-C, and TG, and secondary outcomes included obesity, current smoking, monthly alcohol use, poor self-rated health, and strength exercise (≥ 2 days/week); all outcomes were assessed overall and by sex.

### 4. Data collection

Data were collected from March 9 to April 20, 2025, at the Raphael Nanum Homeless Clinic in Seoul. Recruitment notices were posted on the clinic bulletin board with approval from the clinic administration. Eligible participants were poor urban adults aged ≥ 60 years who visited the clinic during the study period.

For external comparisons, comparator data were obtained from the 2023 KNHANES public-use dataset by extracting adults aged ≥ 60 years. Because 2023 KNHANES includes multiple survey components (health interview, examination, and laboratory measures), analytic denominators differ across indicators; therefore, the 2023 KNHANES denominator used for each outcome was reported in the corresponding comparison tables, and the extraction flow is summarized in Figure 1.

A structured questionnaire was administered at the clinic’s reception area. Researchers explained the study purpose and procedures and responded to participants’ questions. Participants completed the questionnaire individually to ensure independent responses. Completion required approximately 10~15 minutes, and a small gift was provided afterward. For participants who consented to blood testing, fasting blood samples were collected according to standard laboratory protocols, and the results were used for analysis.

### 5. Data analysis

Data were analyzed using IBM SPSS Statistics 25.0 (IBM Corp., Armonk, NY, USA). Descriptive statistics (frequencies, percentages, means, and standard deviations) were calculated for the general characteristics and main variables. General characteristics were compared between the Raphael Nanum Homeless Clinic sample and the 2023 KNHANES comparator group (adults aged ≥ 60 years) using independent t-tests for continuous variables and chi-square tests or two-proportion z-tests for categorical variables, as appropriate. Independent t-tests were used to compare physiological health indicators (total cholesterol, LDL-C, and TG levels) between the study sample and the 2023 KNHANES older adult population. For health indicators (obesity, smoking, alcohol use, poor self-rated health, and strength exercise), two-proportion z-tests (two-tailed) were used to examine differences between groups. All analyses were two-tailed with a significance level of *p* < .050, and results were reported with *p*-values.

### 6. Ethical considerations

This study was approved by the Institutional Review Board (IRB) of Kangwon National University (IRB No: KWNUIRB-2024-12-003-001). Participant recruitment was conducted by independent research staff with no clinical relationship to the subjects to minimize coercion or undue influence. Researchers explained the study objectives, procedures, potential risks and benefits, voluntary participation, right to withdraw, and confidentiality measures. The participants were given 10–15 minutes to review the consent form before providing written informed consent.

Eligibility was confirmed using de-identified clinic records of Raphael Nanum Homeless Clinic visits. These records were used solely to verify prior visits, and no personally identifiable information was included in the study dataset. The sociodemographic information collected included sex, age, education, religion, income status, monthly income, housing status, cohabitation with family, the status of basic livelihood security benefits, and the type of health insurance. All data were anonymized, and identifiers were removed before analysis. Data were securely managed under strict confidentiality and used exclusively for research purposes.

## RESULTS

### 1. General characteristics

Table 1 compares general characteristics between the Raphael Nanum Homeless Clinic sample (n = 60) and adults aged ≥ 60 years in the 2023 KNHANES. The clinic participants were older (79.92 ± 5.61 vs 69.69 ± 6.40 years, *p* < .001) and predominantly men (80.0% vs. 43.9%, *p* < .001). Educational attainment did not differ significantly between groups (*p* = .488). Monthly income was substantially lower in the clinic sample (88.22 ± 84.91 vs. 356.30 ± 329.19 [10,000 KRW, *p* < .001). Housing ownership was reported by 90.0% of the clinic participants compared with 25.2% in the 2023 KNHANES (*p* < .001), and living alone was more common in the clinic sample (45.0% vs. 24.2%, *p* < .001). Beneficiaries of basic livelihood security were also more prevalent in the clinic sample (36.7% vs. 8.7%, *p* < .001). Health insurance distributions differed between groups (*p* < .001): 56.7% had community-based insurance, 13.3% workplace-based insurance, 8.3% received medical aid, and 21.7% were categorized as unknown (not available in 2023 KNHANES). Self-rated health was poorer among clinic participants (poor: 46.7% vs. 24.1%, *p* < .001). Current smoking tended to be lower in the clinic sample (3.3% vs. 10.6%, *p* = .054). Monthly alcohol use was significantly lower in the clinic sample (31.7% vs. 53.0%, *p* < .001), while strength exercise (≥ 2 days/week) did not differ between groups (26.7% vs. 25.3%, *p* = .770). Mean BMI (24.22 ± 2.51 vs. 23.99 ± 3.22 kg/m², *p* = .482) and waist circumference (87.96 ± 12.20 vs. 85.92 ± 9.41 cm, *p* = .204) were not significantly different between groups.

### 2. Comparison of physiological health indicators

In Table 2, compared with age-matched peers in the 2023 KNHANES, the urban poor older adults examined in this study displayed a distinctive lipid profile. Mean total cholesterol was virtually identical overall (176.52 ± 43.21 mg/dL vs. 174.95 ± 45.07 mg/dL), yet stratified analyses showed significantly higher levels in both men (174.10 ± 41.35 mg/dL vs. 168.21 ± 42.83 mg/dL; t = 2.32, *p* = .020) and women (186.17 ± 50.80 mg/dL vs. 180.28 ± 46.08 mg/dL; t = 2.06, *p* = .040). LDL cholesterol was elevated overall (105.20 ± 38.68 mg/dL vs. 95.03 ± 33.19 mg/dL; t = 2.02, *p* = .048), with pronounced increases among men (104.00 ± 37.68 mg/dL vs. 90.50 ± 38.25 mg/dL; t = 5.87, *p* < .001) and women (110.00 ± 45.32 mg/dL vs. 98.40 ± 28.40 mg/dL; t = 4.76, *p* < .001). In contrast, TG trended lower overall and were significantly lower in both sexes (men 117.15 ± 46.13 mg/dL vs. 132.22 ± 118.25 mg/dL, t = −2.79, *p* = .005; women 110.00 ± 40.66 mg/dL vs. 116.88 ± 67.65 mg/dL, t = −2.09, *p* = .037), even though the overall difference was not statistically significant (115.72 ± 44.06 mg/dL vs. 123.43 ± 92.98 mg/dL; t = −1.28, *p* = .205). These findings suggest that, despite similar overall cholesterol levels, urban poor older adults exhibit higher atherogenic LDL cholesterol and lower TG levels relative to their national peers, highlighting an imbalanced lipid profile that may increase cardiovascular risk.

### 3. Comparison of key health indicators

In Table 3, key health indicators were compared between study participants and age‑matched peers in 2023 KNHANES. Obesity (BMI ≥ 25 kg/m²) was more prevalent in the study group overall (50.0 % vs. 35.0 %; z = 2.32, *p* = .020), driven chiefly by men (52.1 % vs. 31.5 %; z = 2.23, *p* = .026), whereas women showed no significant difference (41.7 % vs. 37.7 %; z = 0.24, *p* = .810). Current smoking was markedly less common among study participants (3.3 % vs. 10.6 %; z = −3.10, *p* = .002); men reported a significant reduction (4.2 % vs. 21.4 %; z = −2.51, *p* = .012), while the difference among women was not significant (0.0 % vs. 2.3 %; z = −0.53, *p* = .596). Monthly alcohol consumption (≥ 1/month) was also substantially lower (31.7 % vs. 53.0 %; z = −3.48, *p* < .001), with significant reductions in both men (33.3 % vs. 68.3 %; z = −3.19, *p* = .001) and women (25.0 % vs. 40.6 %; z = −2.11, *p* = .035). Poor self‑rated health was more frequent in the study population overall (46.7 % vs. 24.6 %; z = 2.69, *p* = .007) and particularly among men (45.8 % vs. 20.3 %; z = 2.70, *p* = .007), but the difference was not significant among women (50.0 % vs. 28.0 %; z = 1.43, *p* = .153). Strength exercise (≥ 2 days/week) showed no significant difference overall (26.7 % vs. 25.3 %; z = 0.21, *p* = .834) or by sex (men: 29.2 % vs. 35.9 %; z = −0.57, *p* = 0.570; women: 16.7 % vs. 16.7 %; z = 0.00, *p* = 1.000).

Table 4 presents sex-stratified comparisons using absolute counts; therefore, the z and *p* values may differ from those in Table 3. Among men (n = 48), obesity was higher in the study sample (52.1%) than in KNHANES (31.5%; n = 4,793; z = 2.85, *p* = .004), whereas obesity prevalence among women (n = 12) did not differ (41.7% vs. 37.7%; z = 0.28, *p* = .779). Current smoking was markedly lower among men (4.2% vs. 21.4%; n = 2,515; z = −5.72, *p* < .001), and no women reported smoking (0.0% vs. 2.3%; z = −0.53, *p* = .596). Monthly alcohol consumption was significantly lower in men (33.3% vs. 68.3%; z = −5.10, *p* < .001), while the difference among women was not significant (25.0% vs. 40.6%; z = −1.25, *p* = .213). Poor self-rated health was more frequent among men (45.8% vs. 20.3%; z = 3.49, *p* < .001) but did not reach significance among women (50.0% vs. 28.0%; z = 1.52, *p* = .129). Participation in strength exercise (≥ 2 days/week) did not differ significantly for men (29.2% vs. 35.9%; z = −1.01, *p* = .311) or women (16.7% vs. 16.7%; z = 0.00, *p* > .999).

## DISCUSSION

This study compared key health indicators in poor, older urban adults with those of the general elderly population (aged ≥ 60 years) using data from the 2023 KNHANES. The findings showed that poor, older urban adults exhibited a higher prevalence of obesity (driven largely by the men in the sample), while their rates of current smoking and monthly alcohol consumption were significantly lower than those of the general population. The proportion of participants reporting poor self-rated health was nearly twice as high in the poor group, reflecting worse perceived health status, and participation in strength exercises was low in both groups with no significant difference. In terms of physiological measures, the poor, older adults demonstrated an imbalanced lipid profile notably elevated LDL cholesterol levels (with slightly higher total cholesterol in stratified analyses) and significantly lower triglyceride levels relative to their national peers. These patterns underscore substantial socioeconomic health disparities among the elderly. Such results suggest that accumulated economic difficulties in old age can negatively affect health behaviors and chronic disease management [11,19].

Sex-specific comparisons further illustrated distinct patterns. Among men, the poor urban group had a significantly higher obesity prevalence and a much greater frequency of poor self-rated health compared to their national counterparts, despite markedly lower smoking and alcohol use. Among women, by contrast, obesity prevalence (and other indicators such as strength exercise) did not differ significantly from the general female population. Smoking was negligible in both poor and general older women, and while poor women reported somewhat lower alcohol consumption and a higher proportion with poor health than national figures, these differences were not statistically significant.

Compared with the national data, the poor, older urban adults in this study showed markedly lower smoking and drinking rates. Nationally, smoking and alcohol use remain relatively common among older adults, particularly among men, whereas such behaviors were infrequent in our study population. These findings indicate that lifestyle patterns among the poor urban elderly differ substantially from national averages. Overall, these gender differences may reflect sociocultural influences as well as the unequal impact of socioeconomic conditions on health across men and women [20,21]. In particular, socioeconomic gradients in smoking have been documented in Korea, with household- and area-level income associated with smoking status, which may partly explain differential patterns by gender and social context [22].

The worse health status of poor, older urban adults compared with the general elderly population is consistent with findings from both domestic and international studies. Korea’s elderly poverty rate remains one of the highest among OECD countries (approximately 45%~50%) [11,21], contributing to a higher prevalence of chronic diseases and lower overall health levels in this population. Kim et al. [11] also reported that older adults receiving medical aid showed a higher prevalence of multiple chronic conditions (including depression, cardiovascular and respiratory diseases) compared with insured elderly people, highlighting marked health disparities in extremely poor populations.

Of particular note, the proportion of participants reporting poor subjective health status was significantly higher in the poor, older urban group (46.7% vs. 24.6% in the national data). This disparity was especially pronounced in men – nearly half of poor men rated their health as poor, compared to about one-fifth of men in the general elderly – whereas the difference was not statistically significant among women. Low-income elderly people are generally more likely to self-rate their health as “poor,” and Byun [3] found that lower-income older adults were significantly less likely to report good health. Moreover, when depressive symptoms, nutritional deficiencies, and limitations in daily activities coexist, subjective health declines further [3]. These observations suggest that nutritional factors and mental health problems may have been interacting factors among the poor, older urban adults in this study. Chronic undernutrition and social isolation may contribute to both underweight and obesity, creating a dual burden that exacerbates health disparities [23,24].

The higher prevalence of obesity observed in this study compared with the general elderly population, particularly among men, suggests a pattern of nutritional imbalance rather than simple overnutrition. This finding aligns with previous research indicating that socioeconomic disadvantage can increase the risk of obesity through poor dietary quality and limited access to healthy foods [23]. In urban poor environments, older adults often rely on inexpensive, calorie-dense foods with low nutritional value, which may contribute to excessive body weight and metabolic complications. Such obesity related to poor diet quality can coexist with nutritional deficiencies, representing a form of “hidden hunger” that leads to higher risks of chronic disease and poorer perceived health.

Notably, both the poor and general elderly groups exhibited extremely low rates of strength exercise, reflecting severe physical inactivity consistent with previous studies. In our sample, only about one-quarter of the poor older adults practiced muscle-strengthening exercises at least two days a week – a level comparable to their age-matched peers nationally and indicative of a widespread lack of exercise in later life. Lee et al. [19] similarly reported that only around 7 % of Koreans aged ≥ 65 met recommended physical activity guidelines, with mortality risks from physical inactivity disproportionately higher among low-income groups.

Regular physical activity reduces cardiovascular disease and all-cause mortality in older adults, with even greater benefits observed in socioeconomically disadvantaged groups [19]. Nevertheless, social vulnerabilities restrict opportunities for exercise due to financial constraints and limited access to facilities, often excluding disadvantaged older adults from these health benefits [19,25]. Our findings underscore that inadequate exercise participation among low-income elderly people remains a major concern. A lack of physical activity contributes to obesity and accelerates functional decline, ultimately lowering quality of life. Prior studies have found that obese older adults have poorer health-related quality of life than those with normal weight, with stronger negative effects observed among women [21,23]. Thus, physical inactivity may exacerbate obesity and impair daily functioning in poor, older adults – particularly in women – further undermining their quality of life.

Deficiencies in chronic disease management were also noted. Participants had elevated LDL cholesterol levels compared to national averages (despite similar total cholesterol and even lower TG), suggesting areas of inadequate dyslipidemia management. Low-income older adults are at greater risk of non-adherence to treatment, and Han et al. [26] reported low continuity of care and frequent irregular medication use in this population. These findings reflect the reality that economic difficulties and limited healthcare access can reduce treatment adherence. Despite Korea’s expansion of healthcare coverage, poor, older adults remain vulnerable in preventive care and lifestyle modification [11]. Thus, while hospital utilization may be possible, structural barriers still hinder health promotion and the long-term management of chronic conditions.

Overall, health disparities among poor, older urban adults reflect not only individual lifestyle factors but also broader socioeconomic determinants [27]. Poverty contributes to cumulative health risks through undernutrition, chronic stress, limited access to health information, and social isolation, leading to more severe disease and a poorer prognosis [11]. In particular, weakened family structures and the lack of social support exacerbate mental health problems and limit self-care capacity among poor elderly people [24]. This study thereby confirms the overall health vulnerability of poor, older urban adults, aligning with existing research that reports significant health inequalities within the elderly Korean population [11,21].

Some findings in the current study differed from prior expectations. We observed a higher obesity prevalence in poor older men relative to the general elderly male population, contradicting suggestions that low-income elderly men might have lower obesity due to more manual labor [20]. In contrast, although a higher obesity risk among low-income women has been noted in previous studies [20], our data did not show a significant obesity increase in poor women. Moreover, differences in total cholesterol levels between the poor and general groups were relatively small, which may reflect improved treatment and management of hyperlipidemia thanks to Korea’s universal health coverage expansion [26]. However, pronounced disparities were evident in other health-related indicators: for example, our poor group had substantially different lifestyle patterns – including much lower smoking and drinking rates – compared to national averages, indicating unique health behavior adaptations in this extremely low-income population.

The expansion of healthcare utilization alone is insufficient to address health disparities among poor, older urban adults. Public health interventions to improve health behaviors are essential. Policy implications include strengthening home-visit and mobile health services [27], expanding low-cost community exercise programs [19,28], enhancing nutritional support and dietary education [24], improving mental health and social support systems [27,29], and developing gender-specific health-promotion strategies [21].

In conclusion, health inequalities among poor, older urban adults are related to structural socioeconomic determinants and not solely individual behaviors, and urgent, multifaceted policy interventions are needed. Such efforts will contribute to sustaining Korea’s super-aged society and improve the quality of life in later years [11,21].

This study should be interpreted in light of several limitations. First, although the required sample size was estimated a priori, the number of participants included in the final analyses was limited. Some variables had missing responses or incomplete measurements, and analytic denominators therefore varied by indicator. This reduced statistical power for detecting group differences and increases the possibility of Type II error, particularly for outcomes with low prevalence. Accordingly, nonsignificant findings should be interpreted cautiously as they may reflect limited power rather than the absence of true differences.

Second, the study sample was recruited from a free clinic serving medically vulnerable urban older adults, which may introduce selection bias and limits generalizability to the broader population of low-income older adults. The results may best represent older adults who actively seek care in a free-clinic setting and may not be directly applicable to those who do not access such services.

Future studies should recruit larger samples using multi-site sampling (e.g., multiple free clinics and community-based outreach sites) to enhance representativeness and allow adequately powered subgroup analyses (e.g., by sex and age strata). Prospective designs and standardized protocols to minimize missingness across measurements would also strengthen inference and improve external validity.

## CONCLUSION

This study demonstrated that poor, older urban adults exhibit distinct and multidimensional health disparities compared with the general elderly population in Korea. Physiologically, they showed higher LDL cholesterol levels and lower triglyceride concentrations, indicating an unfavorable metabolic profile. Behaviorally, the group was characterized by a higher prevalence of obesity—particularly among men—along with markedly lower rates of smoking and alcohol use compared with national data. In addition, nearly half of participants rated their health as poor, and engagement in strength exercise was consistently low, underscoring limited physical activity regardless of sex.

These findings highlight that the health of poor, older urban adults is compromised not only by biological risk factors but also by the cumulative effects of socioeconomic deprivation, restricted access to nutritious food, inadequate opportunities for exercise, and psychosocial vulnerability. Addressing these inequities requires comprehensive and sustainable public health strategies that extend beyond healthcare utilization—such as expanding community-based health promotion programs, strengthening nutritional and social support, and implementing gender-responsive interventions.

Ultimately, reducing health inequalities among poor, older urban adults is critical to ensuring healthy and dignified aging within Korea’s rapidly aging society.

## Figures and Tables

**Table 1.** Comparison of General Characteristics between Study Participants and Adults (Aged ≥ 60 Years) in the 2023 KNHANES (N = 60)

Variables	Categories	Study participants (n = 60)	KNHANES (≥ 60 years, n=2,522)	t or χ² or z	*p*
Sex	Men	48 (80.0)	1,108 (43.9)	29.39	< .001
	Women	12 (20.0)	1,414 (56.1)		
Age (years)		79.92 ± 5.61	69.69 ± 6.40	13.91	< .001
Education	≤ Elementary school	23 (38.3)	888 (36.8)	1.43	.488
	Middle school	15 (25.0)	478 (19.8)		
	≥ High school	22 (36.7)	1,046 (43.4)		
Monthly income (10,000 KRW)		88.22 ± 84.91	356.30 ± 329.19	–20.99	< .001
Housing ownership	Yes	54 (90.0)	635 (25.2)	122.51	< .001
	No	6 (10.0)	1,886 (74.8)		
Living alone	Yes	27 (45.0)	610 (24.2)	12.56	< .001
	No	33 (55.0)	1,912 (75.8)		
Basic livelihood security recipient	Yes	22 (36.7)	218 (8.7)	51.25	< .001
	No	38 (63.3)	2,302 (91.3)		
Health insurance	Community-based	34 (56.7)	947 (37.6)	27.80	< .001
	Workplace-based	8 (13.3)	1,395 (55.3)		
	Medical aid	5 (8.3)	180 (7.1)		
	Unknown	13 (21.7)	-		
Self-rated health	Good	8 (13.3)	618 (27.3)	17.23	< .001
	Fair	24 (40.0)	1,101 (48.6)		
	Poor	28 (46.7)	545 (24.1)		
Current smoking	Yes	2 (3.3)	272 (10.6)	–1.93	.054
	No	58 (96.7)	2,149 (89.4)		
Alcohol use (≥ 1 drink/month)	Yes	19 (31.7)	1,296 (53.0)	–3.32	< .001
	No	41 (68.3)	1,134 (47.0)		
Strength exercise (≥ 2 days/week)	Yes	16 (26.7)	565 (25.3)	0.29	.770
	No	44 (73.3)	1,694 (74.7)		
Body mass index (kg/m²)		24.22 ± 2.51	23.99 ± 3.22	0.70	.482
Waist circumference (cm)		87.96 ± 12.20	85.92 ± 9.41	1.29	.204

Values are presented as the mean ± standard deviation or n (%). 2023 KNHANES values were derived from 2023 KNHANES public-use dataset for adults aged ≥ 60 years; analytic denominators vary by variable because of missing or inapplicable responses. Test statistics are reported as t, χ², or z. For health insurance, the “Unknown” category in the study sample was excluded from the chi-square test because no comparable category was available in the 2023 KNHANES. KNHANES = Korea National Health and Nutrition Examination Survey; KRW = Korean won.

**Table 2.** Comparison of Physiological Health Indicators between Study Participants and Adults (Aged ≥ 60 Years) in the 2023 KNHANES

Variables	Study participants (Total)	KNHANES (Total)	t (*p*)	Study participants (Men)	KNHANES (Men)	t (*p*)	Study participants (Women)	KNHANES (Women)	t (*p*)
Total cholesterol (mg/dL)	176.52 ± 43.21	174.95 ± 45.07	0.28 (.782)	174.10 ± 41.35	168.21 ± 42.83	2.32 (.020)	186.17 ± 50.80	180.28 ± 46.08	2.06 (.040)
LDL-C (mg/dL)	105.20 ± 38.68	95.03 ± 33.19	2.02 (.048)	104.00 ± 37.68	90.50 ± 38.25	5.87 (<.001)	110.00 ± 45.32	98.40 ± 28.40	4.76 (<.001)
TG (mg/dL)	115.72 ± 44.06	123.43 ± 92.98	–1.28 (.205)	117.15 ± 46.13	132.22 ± 118.25	–2.79 (.005)	110.00 ± 40.66	116.88 ± 67.65	–2.09 (.037)

Values are presented as the mean ± standard deviation. KNHANES = Korea National Health and Nutrition Examination Survey; LDL = Low density lipoprotein-cholesterol; TG = Triglycerides.

**Table 3.** Comparison of Key Health Indicators between Study Participants and Adults (Aged ≥ 60 Years) in the 2023 KNHANES

Indicator	Study participants Total (%)	KNHANES Total (%)	z (*p*)	Study participants Men (%)	KNHANES Men (%)	z (*p*)	Study participants Women (%)	KNHANES Women (%)	z (*p*)
Obesity (BMI ≥25 kg/m²)	50.0	35.0	2.32 (.020)	52.1	31.5	2.23 (.026)	41.7	37.7	0.24 (.810)
Current smoker	3.3	10.6	−3.10 (.002)	4.2	21.4	−2.51 (.012)	0.0	2.3	−0.53 (.596)
Alcohol consumption (≥ 1 drink/month)	31.7	53.0	−3.48 (<.001)	33.3	68.3	−3.19 (.001)	25.0	40.6	−2.11 (.035)
Poor self-rated health	46.7	24.6	2.69 (.007)	45.8	20.3	2.70 (.007)	50.0	28.0	1.43 (.153)
Strength exercise (≥ 2 days/week)	26.7	25.3	0.21 (.834)	29.2	35.9	−0.57 (.570)	16.7	16.7	0.00 (> .999)

KNHANES = Korea National Health and Nutrition Examination Survey; BMI = Body mass index.

**Table 4.** Sex-stratified Comparison of Key Health Indicators between Study Participants and Adults (Aged ≥ 60 Years) in the 2023 KNHANES (N = 60)

Indicator	Group	Study participants n		KNHANES		z	*p*
			(%)	n	(%)		
Obesity (BMI ≥25 kg/m²)	Men	48	(52.1)	4,793	(31.5)	2.85	.004
	Women	12	(41.7)	6,164	(37.7)	0.28	.779
Current smoker	Men	48	(4.2)	2,515	(21.4)	−5.72	< .001
	Women	12	(0.0)	3,248	(2.3)	−0.53	.596
Alcohol consumption	Men	48	(33.3)	2,521	(68.3)	−5.10	< .001
	Women	12	(25.0)	3,251	(40.6)	−1.25	.213
Poor self-rated health	Men	48	(45.8)	1,001	(20.3)	3.49	< .001
	Women	12	(50.0)	1,263	(28.0)	1.52	.129
Strength exercises (≥ 2 days/week)	Men	48	(29.2)	3,223	(35.9)	−1.01	.311
	Women	12	(16.7)	3,071	(16.7)	0.00	> .999

Sex-stratified analyses were conducted using absolute counts. KNHANES = Korea National Health and Nutrition Examination Survey.

## Data Availability

The datasets are not publicly available but are available from the corresponding author upon reasonable request.
